# Genome-wide linkage search for cancer susceptibility loci in a cohort of non BRCA1/2 families in Sri Lanka

**DOI:** 10.1186/s13104-022-06081-5

**Published:** 2022-06-02

**Authors:** Prabhavi Wijesiriwardhana, Anthony M. Musolf, Joan E. Bailey-Wilson, T. Kalum Wetthasinghe, Vajira H. W. Dissanayake

**Affiliations:** 1 Department of Medical Laboratory Science, Faculty of Allied Health Sciences, University of Ruhuna, Galle, Sri Lanka; 2 Computational and Statistical Genomics Branch, National Human Genome Research Institute, National Institutes of Health, Baltimore, MD 21224 USA; 3 Human Genetics Unit, Faculty of Medicine, University of Colombo, Colombo, Sri Lanka

**Keywords:** Familial cancer, Hereditary cancers, Linkage analysis, Genotyping, Whole exome sequencing

## Abstract

**Objective:**

Although linkage studies have been utilized for the identification of variants associated with cancer in the world, little is known about their role in non *BRCA1/2* individuals in the Sri Lankans. Hence we performed linkage analysis to identify susceptibility loci related to the inherited risk of cancer in a cohort of Sri Lankans affected with hereditary breast cancer. The Illumina global screening array having 654,027 single nucleotide polymorphism markers was performed in four families, in which at least three individuals within third degree relatives were affected by breast cancer. Two-point parametric linkage analysis was conducted assuming disease allele frequency of 1%. Penetrance was set at 90% for carriers with a 10% phenocopy rate.

**Results:**

Thirty-one variants exhibited genome-wide suggestive HLODs. The top overall HLOD score was at rs1856277, an intronic variant in *MYO16* on chromosome 13. The two most informative families also suggested several candidate linked loci in genes, including *ERAP1*, *RPRM*, *WWOX*, *CDH1*, *EXOC1, HUS1B*, *STIM1* and *TUSC1*. This study provides the first step in identifying germline variants that may be involved in risk of cancer in cancer-aggregated non-*BRCA1/2* families from the understudied Sri Lankan population. Several candidate linked regions showed suggestive evidence of linkage to cancer risk.

**Supplementary Information:**

The online version contains supplementary material available at 10.1186/s13104-022-06081-5.

## Introduction

Inheritance of cancers among individuals in high risk families can be explained by significant familial aggregation of high, moderate or low penetrance genetic variants in cancer predisposing genes (CPGs) that are transmitted down the generations in each family [1]. Breast cancer has become one of the leading causes of deaths, worldwide [2]. It is estimated that 5–10% of breast cancer patients have a hereditary predisposition and are harboring germline high, moderate or low risk variants in CPGs [3]. Many studies have revealed that a significant proportion of families with many affected cases are not associated with variants in known CPGs such as *BRCA1* and *BRCA2* [4]. However, families negative after breast cancer diagnostics rarely fulfill breast cancer screening criteria, mostly because of a later onset or reduced penetrance [5]. It is also possible that there are further loci conferring more substantial risk that could be detected. In such instances Genome Wide Association Studies (GWAS) have been used to find common genetic variants associated with individually small but additive risk to develop breast cancer in families that are unlikely to be segregating *BRCA1* and *BRCA2* pathogenic variants [6]. So far identified cancer susceptibility genes can only explain up to 5% of all cases, while familial clustering is seen in other cancer affected cases who have been identified as variant negative [7]. There is however, a dearth in the knowledge and understanding of the genes that are responsible for the variant negative affected cases who exhibit evidence of hereditary cancer predisposition among their family members in the Sri Lankan population. This deficiency in knowledge has also resulted in sub optimal management of individuals who are at risk of inherited cancer syndromes.

This is the first linkage analysis study conducted in the families affected with cancer in the Sri Lankan population. A genome wide linkage (GWL) scan was performed using data from 48 individuals from 4 cancer families, aiming to evaluate the possibility of identifying susceptibility loci conferring breast and other cancer predisposition.

## Main text

### Materials and methods

Index cases recruited into this study were women affected with breast cancer who had a family history of breast cancer but who have been identified as having no variants in CPGs (Additional file 1: Figures S1–S4). They were also negative for multiplex ligation-dependent probe amplification assay (MLPA). Three of the 4 index cases in the families studied had an age at diagnosis of breast cancer of less than 50 years (Additional file 2: Table S1). Each index case also had at least 2 relatives who were also affected with breast cancer. In two of the families, multiple family members also were affected with cancer at other sites as well. A total of 21 family members were diagnosed with cancer in addition to the index cases. The index cases and 44 of their affected and unaffected relatives enrolled in the study and provided biospecimens for genotyping (Additional file 2: Table S2).

#### SNP genotyping

Genotyping of 48 individuals was performed at the Australian Genomic Research Facility (AGRF) (Melbourne, Australia) using the Illumina Global Screening Array which has 654,027 single nucleotide polymorphism (SNP) markers on the array. Quality assessment of the samples was performed by QuantiFluor. The genome-wide content was selected for high imputation accuracy at minor allele frequencies of  > 1% across all 26 1000 genomes project populations.

#### Quality control

The software program PLINK [8] was used to perform quality control on the data. We removed all monomorphic variants and variants that were not genotyped in at least 95% of the subjects. Variants with Mendelian inconsistencies were removed from the offending family. Identity-by-descent (IBD) calculations were used to confirm all familial relationships within the four pedigrees. The final dataset contained 236,142 total variants for 69 individuals across the four families, 44 of which had genotype data. Out of the 44 genotyped individuals, 18 were affected with cancer.

#### Parametric linkage analysis

We performed two-point parametric linkage analysis on this data using Merlin [9], which utilizes the well-known Lander-Green algorithm to calculate linkage. We assumed an autosomal dominant model (mode of inheritance was inferred from the pedigrees) with a disease allele frequency of 1%. Penetrance was set at 90% for carriers with a 10% phenocopy rate. LOD scores were calculated for each of the four individual families and heterogeneity LOD (HLOD) scores were calculated across families. All variants were annotated using wANNOVAR [10, 11]

## Results

**Fig. 1:**
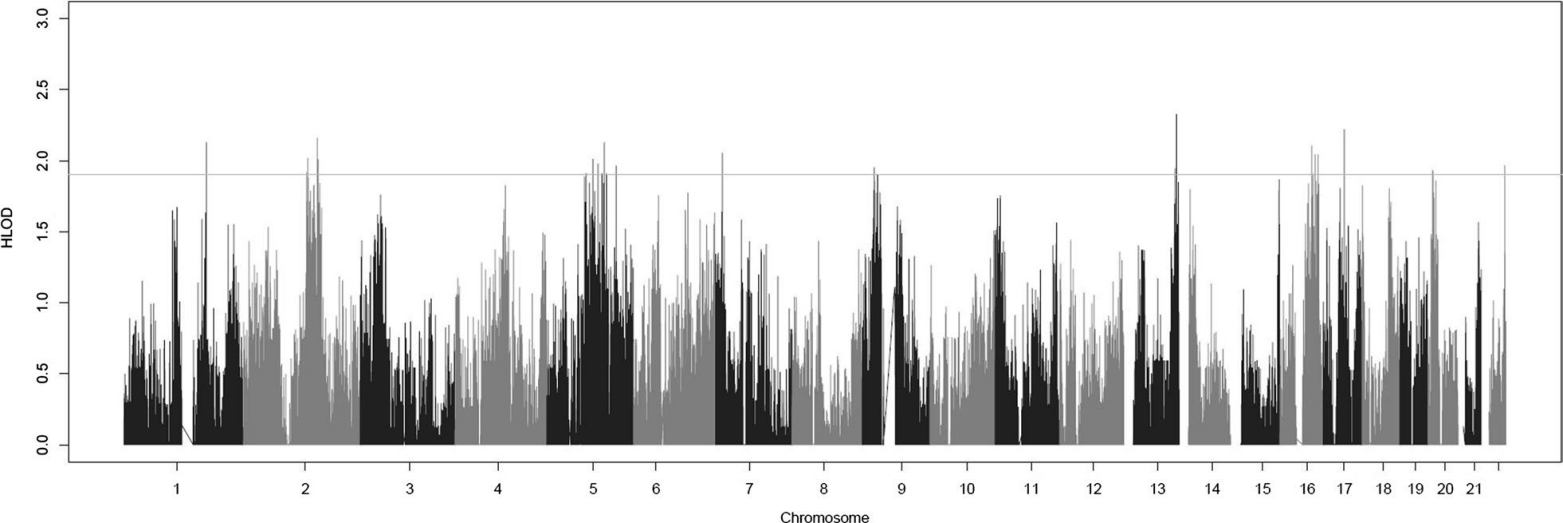
A genome-wide plot of the HLOD scores across the families

**Fig. 2:**
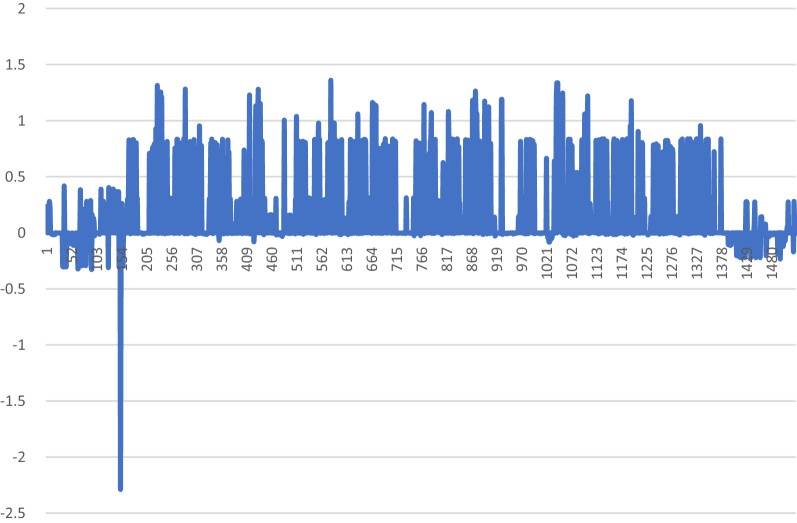
Zoomed-in plot of LOD scores in family 2 on chromosome 6 from 382,507 to 650,645 bp, the region showing evidence of linkage with virtually no negative LOD scores

Thirty-one variants exhibited genome-wide suggestive HLODs (Fig. 1, Table 1). The top overall HLOD score was at rs1856277, an intronic variant in *MYO16* on chromosome 13. There were 13 HLOD scores greater than 2.00. Family 1 was not particularly informative by itself. There was no individual LOD score  > 0.54 and nearly every chromosome had a LOD score of that value (Additional file 2: Table S3, Additional file 1: Figure S5). Thus, nothing of particular interest could be gleaned from this family. The highest LOD score in family 2 was 1.397 at rs12616962, an intronic variant in *KCNJ3* on chromosome 2 (Additional file 2: Table S4, Additional file 1: Figure S6). Chromosome 2 had multiple high LOD scores, in fact the top eight LOD scores in this family were on chromosome 2. However, there were multiple chromosomes (6; 13; 20; 22) that had LOD scores around 1.3. The most intriguing of these results was a peak on chromosome 6 from 382,507 to 650,645 bp (Fig. 2). This peak has almost no negative signal across the region. Most of the very positive LOD scores on chromosome 6 in this family occur in this LOD score peak region and there are no LOD scores more negative than − 0.09 in this region (positive and negative LOD scores very close to zero indicate marker loci with no information content for linkage). That is a hallmark of true linkage—a long stretch of positive LOD scores with no negative LOD scores. While there are other long stretches of positive LOD scores for this family, all of them have lots of negative LOD scores within those same stretches. Hence, we can’t rule those regions out but can say that this region on chromosome 6 can be identified as a better candidate region to contain a high-risk genetic variant for cancer susceptibility in this family. Family 3 had the highest LOD scores of any of the individual families (Additional file 2: Table S5, Additional file 1: Figure S7). There were three main peaks. The highest peak was on chromosome 9, which had two SNPs with LOD scores approximately equal to 1.8. These are rs1925508, an intergenic SNP between *IZUMO3* and *TUSC1*, and rs10812758 an intronic SNP in *LINGO2*. The second peak was on chromosome 11 which had two SNPs with LOD scores of approximately 1.7. These SNPs are rs11825543, an intronic variant in *STIM1* and rs2071461, an exonic variant in *CSNK2A3*. The last peak was on chromosome 16, which had three SNPs with LOD scores of 1.59. All three SNPs were intergenic variants between *MAF* and *MAFTRR*. WES data were not available for any individuals in this family so the candidate regions could not be interrogated further. Much like family 1, family 4 was not informative on its own. There were over 2,000 SNPs with LOD scores between 0.76 and 0.70 across 18 autosomes (Additional file 2: Table S6, Additional file 1: Figure S8).

**Table 1:** Top linked regions across all the families with the top HLOD score in each region

	CHR	POS	ID	HLOD	FUNC	GENE
1.	13	1,094,516	GSA-rs1856277	2.3254	Intronic	*MYO16*
2.	13	1,094,545	rs58775680	2.2902	Intronic	*MYO16*
3.	17	438,098.4	rs4414533	2.2183	Intronic	*LINC02210-CRHR1*
4.	2	1,540,325	rs892747	2.1588	Intergenic	*ARL6IP6;RPRM*
5.	1	1,719,610	rs10910863	2.1281	Intronic	*DNM3*
6.	5	1,200,491	rs9285894	2.1267	Intergenic	*PRR16;LOC102467226*
7.	16	663,989.3	rs9940044	2.1039	Intergenic	*LINC00922;CDH5*
8.	7	141,367.4	GSA-rs10085723	2.0531	Intergenic	*ETV1;DGKB*
9.	16	731,506	rs11075965	2.0431	Intergenic	*HCCAT5;C16orf47*
10.	16	795,736	GSA-rs78740081	2.0409	Intergenic	*WWOX;MAF*
11.	2	1,343,145	rs13021524	2.0166	Intronic	*NCKAP5*
12.	5	961,424.8	rs62376387	2.0108	Intronic	*ERAP1*
13.	2	1,556,290	GSA-rs12616962	2.0074	Intronic	*KCNJ3*
14.	16	796,487.4	rs7189280	1.9795	Intergenic	*MAF;MAFTRR*
15.	5	1,068,795	exm2270234	1.9775	Intronic	*EFNA5*
16.	23	382,099	rs4826994	1.9728	Intergenic	*NONE;NONE*
17.	23	382,162.5	rs5917575	1.9728	Intergenic	*NONE;NONE*
18.	22	487,296.7	GSA-rs34134485	1.9665	Intergenic	*MIR3201;FAM19A5*
19.	5	1,445,266	rs2400169	1.963	Intergenic	*KCTD16;PRELID2*
20.	23	382,357.9	rs5917584	1.9513	Intergenic	*NONE;NONE*
21.	9	251,777.2	GSA-rs1925508	1.9508	Intergenic	*IZUMO3;TUSC1*
22.	13	1,064,320	rs17501394	1.9457	Intergenic	*LINC00343;LINC00460*
23.	20	100,921.6	GSA-rs6133801	1.9311	ncRNA_intronic	*SNAP25-AS1*
24.	20	108,977.9	rs6108744	1.9236	Intergenic	*LOC101929413;C20orf187*
25.	2	1,545,392	GSA-rs16834032	1.9213	Intergenic	*RPRM;GALNT13*
26.	2	1,317,662	GSA-rs74813861	1.9176	Intronic	*ARHGEF4*
27.	5	819,896.3	rs10072525	1.9099	Intergenic	*ATP6AP1L;MIR3977*
28.	5	1,153,587	rs2662482	1.9086	Intronic	*LVRN*
29.	5	1,245,335	GSA-rs73313556	1.9074	ncRNA_intronic	*LOC101927421*
30.	16	688,224.8	rs8049967	1.9062	Intronic	*CDH1*
31.	5	961,394.6	rs3734016	1.9042	Exonic	*ERAP1*

*CHR* chromosome, *POS* position, *ID* marker identity, *HLOD* highest logarithm of odds, *FUNC* type/function of the variant; *GENE* corresponding gene/genes

## Discussion/conclusion

The present linkage study performed across 4 Sri Lankan non-*BRCA1/2* families ascertained due to a family history of breast cancer, resulted in suggestive evidence for linkage to cancer risk at candidate regions on chromosomes 2, 6, 13 and 22. These results suggest the presence of several putative loci for risk of breast or other cancer. These results suggested that heterogeneity among families could mask linkage signals, especially when the number of families is small. Importantly, two of the families had only breast cancer patients while the two most informative families for linkage had multiple family members also affected with other cancers. Thus, it is not surprising that several different candidate regions are identified in this analysis of all-cancer susceptibility. Looking at the regions with high HLODs across the four families, we find variants in four genes that are particularly intriguing for cancer risk—*ERAP1*, *RPRM*, *WWOX*, and *CDH1*. Low expression of endoplasmic reticulum aminopeptidase 1 (*ERAP1*) gene has been associated with poor clinical outcomes of patients affected with triple negative breast carcinoma [12]. Reprimo gene (*RPRM*) is a potential p53-dependent tumor suppressor gene [13]. The *RPRM* gene has been found to be frequently hypermethylated in several human cancers [14]. Loss of heterozygosity, homozygous deletions, and chromosomal translocations affecting WW domain containing oxidoreductase (*WWOX*) gene has been reported mainly in breast cancer but also including ovarian, esophageal, lung and stomach carcinoma, and multiple myeloma [15]. An intragenic GSA-rs78740081 variant with a 2.0409 of HLOD score in the *WWOX* gene has been identified as a genome wide suggestively linked variant in this cohort. The region on chromosome 16 where E-cadherin (*CDH1*) gene is located is frequently associated with loss of heterozygosity and loss of tumour suppressor function in several cancers, including gastric [16], colorectal [17], breast [18] and ovarian [19]. We have found a genome-wide suggestive linkage to an intronic variant in the 688,224.8 bp position in the chromosome where the *CDH1* gene resides. Family 2 showed top linked variants in multiple genes that are interesting candidates for cancer susceptibility, including *KCNJ3* and *EXOC2*.

Several studies on linkage analysis in non *BRCA1/2* families have been conducted in other populations [20–25]. However, the fact that these findings do not replicate in other populations is not surprising given the uniqueness of this Sri Lankan data set.

There are several strengths and weaknesses of this study. In a heterogeneous disease like cancers, it would be unsurprising to find novel candidate genes and variants in different populations and in different families within a population. Family-based linkage studies such as this are able to utilize the long, linked haplotypes shared by closely related affected individuals, allowing for identification of linked chromosomal regions that may harbor causal variants that might not have been genotyped in this study. Variants that are rare in the general population may also be enriched in individual families ascertained for a strong family history of cancer, particularly early-onset cancers.

In conclusion, this study provides the first step in identifying germline causal variants that may be involved in risk of cancer in cancer-aggregated families from the understudied Sri Lankan population. Several candidate chromosomal regions showed suggestive evidence of linkage to cancer risk.

## Limitations

There are some limitations in this study as well. First, we used a microarray chip, meaning that there were large numbers of variants that were not genotyped. Thus, it is possible that we may have not identified a causal variant in this study, but more likely, a variant(s) that is linked to the casual variant. Even in family 2 where we were able to identify candidate exonic variants in the index case that were within the linked regions, it is possible that a variant that is in this linkage region but not covered by WES is the causal variant. Targeted sequencing of the candidate regions will be needed to elucidate the true causal variants. It is of course also possible that all of these linkage results are false positives because of the relatively small number of biospecimens available on affected family members. The number of patients and their family members who undergo WES testing is very low in Sri Lanka due to the high cost of the diagnostic testing. Out of the very few patients in Sri Lanka who underwent NGS testing, many were found to have at least one SNV in high, moderate and low risk cancer predisposing genes hence we had to exclude them from our study. Only the breast cancer probands who visited our clinics for genetic screening and had negative results in the testing of known cancer syndrome genes were invited to join this study and SNP genotyping was done for the family members who we recruited for the study. We plan to address these issues in future studies. Power can be improved by attempting to enroll more relatives within these four families and by adding more families to the study. We also plan additional studies using multipoint linkage techniques and plan to seek funding for additional genotyping and sequencing of informative family members. A detailed analysis of the phenotypic and clinical characteristics of this cohort in relation to the genotypic results is the subject of a future study.

## Supplementary Information

**Additional file 1: Figure S1.** Family 1. **Figure S2.** Family 2. **Figure S3.** Family 3. **Figure S4.** Family 4. **Figure S5.** Plot of two-point LOD scores for Family 1. **Figure S6.** Plot of two-point LOD scores for Family 2. **Figure S7.** Plot of two-point LOD scores for Family 3. **Figure S8.** Plot of two-point LOD scores for Family 4.

**Additional file 2: Table S1.** Details of the cancer in affected relatives of the breast cancer index patient in each family. **Table S2.** The health status and the number of family members who provided biospecimens for genotyping. **Table S3.** A description of top LOD scores of family 1. **Table S4.** A description of top LOD scores of family 2. **Table S5.** A description of top LOD scores of family 3. **Table S6.** A description of top LOD scores of family 4.

## Data Availability

The datasets used and/or analyzed during the current study are available from the corresponding author on reasonable request.

## Abbreviations

HLOD: Heterogeneity logarithm of odds
LOD: Logarithm of odds
CPG: Cancer predisposing genes
SNP: Single nucleotide polymorphism
WES: Whole exome sequencing
MLPA: Multiplex ligation probe dependent amplification assay
GSA: Global screening array
