# Supplementary material for: Genome-wide linkage search for cancer susceptibility loci in a cohort of non BRCA1/2 families in Sri Lanka
Source: BMC Res Notes. 2022 Jun 2;15:190. doi: 10.1186/s13104-022-06081-5 (PMC9164366; doi:10.1186/s13104-022-06081-5)
Supplement: Supplementary file 1 — Additional file 1: Figure S1. Family 1. Figure S2. Family 2. Figure S3. Family 3. Figure S4. Family 4. Figure S5. Plot of two-point LOD scores for Family 1. Figure S6. Plot of two-point LOD scores for Family 2. Figure S7. Plot of two-point LOD scores for Family 3. Figure S8. Plot of two-point LOD scores for Family 4. [file 13104_2022_6081_MOESM1_ESM.docx]

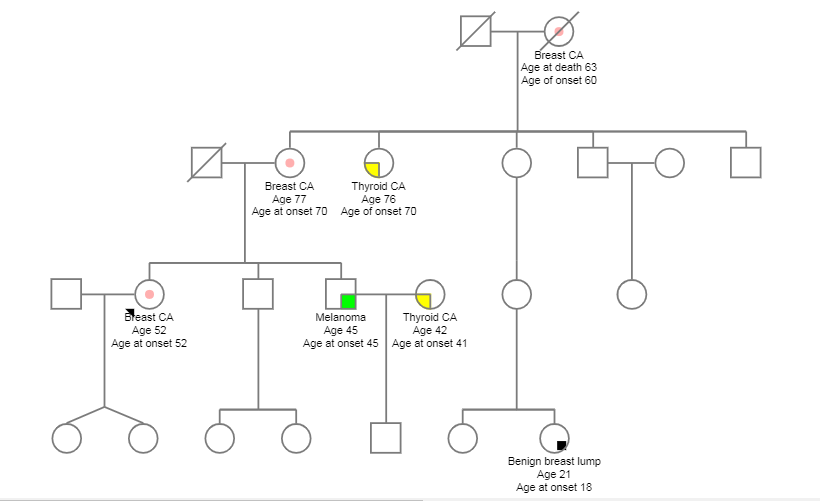

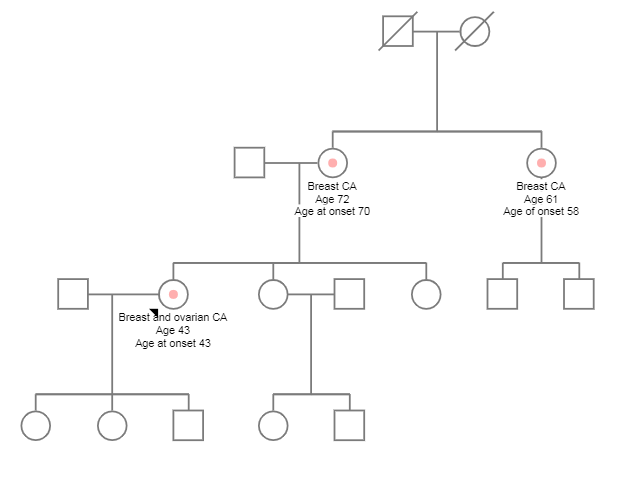


Figure S2: Family2

Figure S1: Family1

* blood was drawn

* blood was drawn

*

*

*

*

*

*

*

*

*

*

*

*

*

*

*

*

*

*

*

*

*

*

*


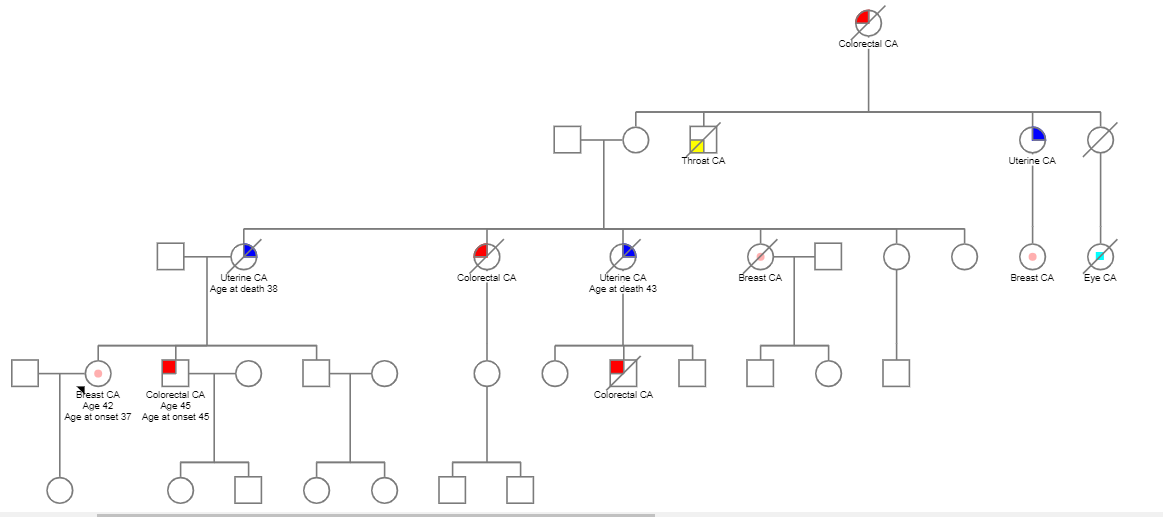


*

*

*

*

*

*

*

*

*

*

*

*

*

*

*

*

*

*

*

Figure S3 :Family3

* blood was drawn


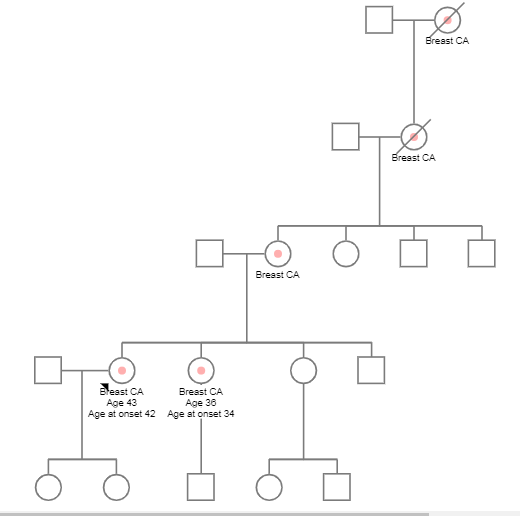


*

*

Figure S4: Family4

* blood was drawn

*

*

*

*


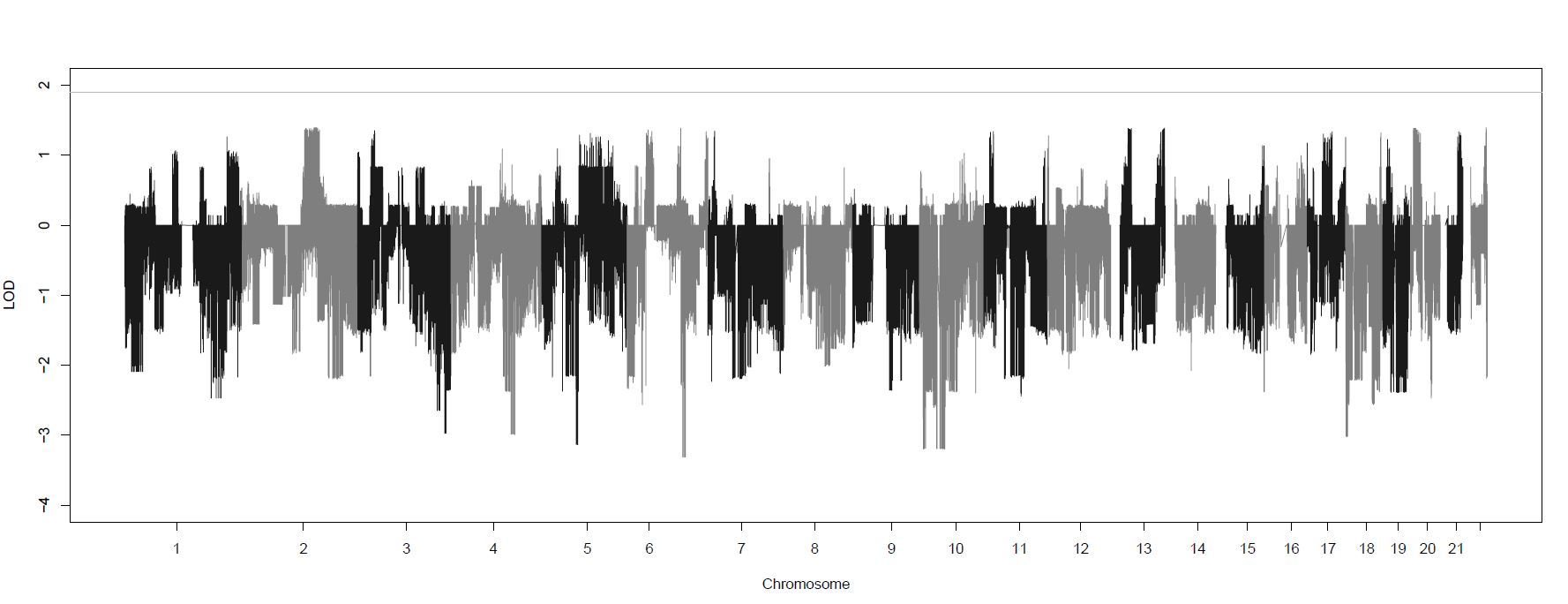

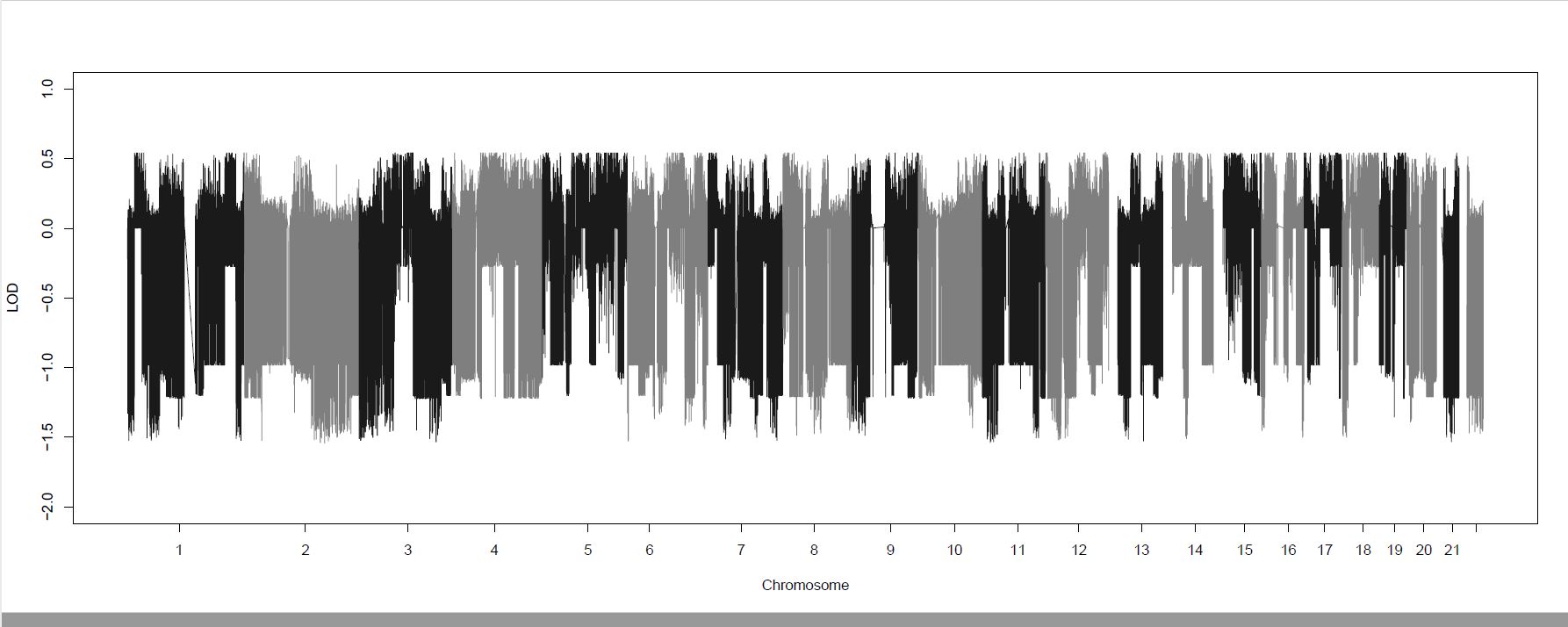


Figure S5: Plot of two-point LOD scores for Family 1

Figure S6: Plot of two-point LOD scores for Family 2


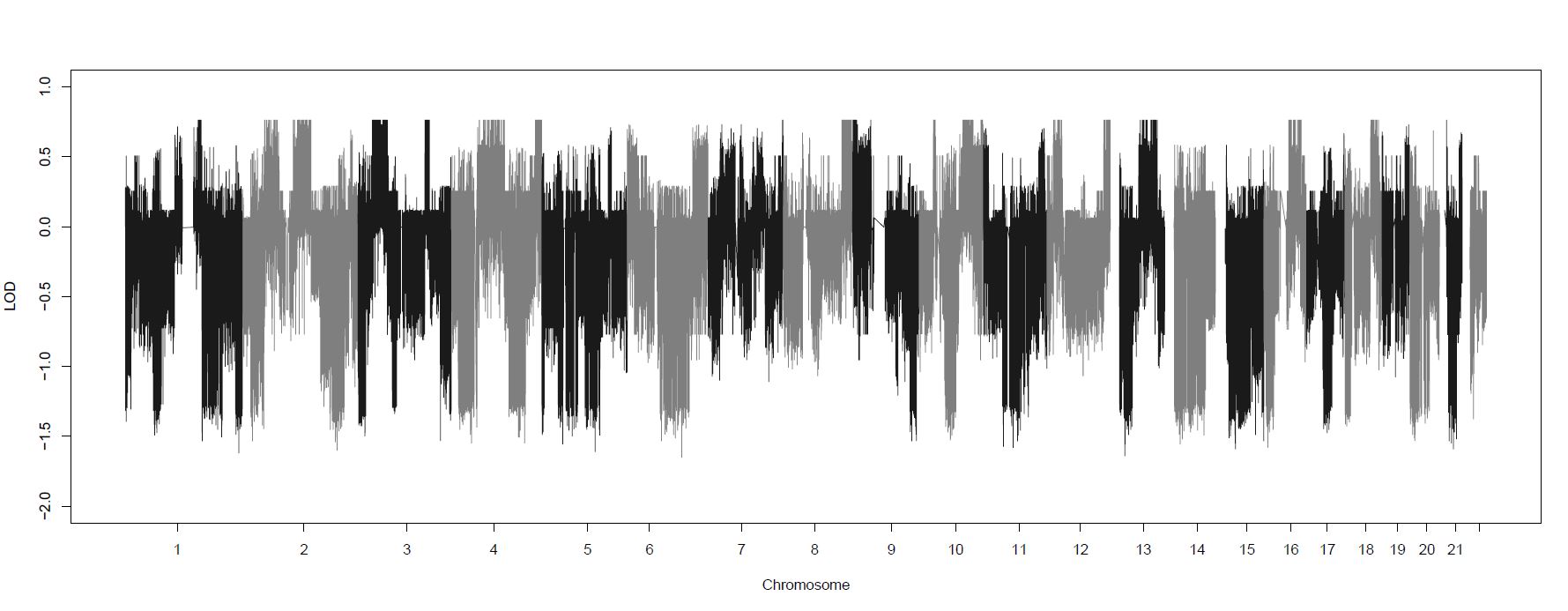

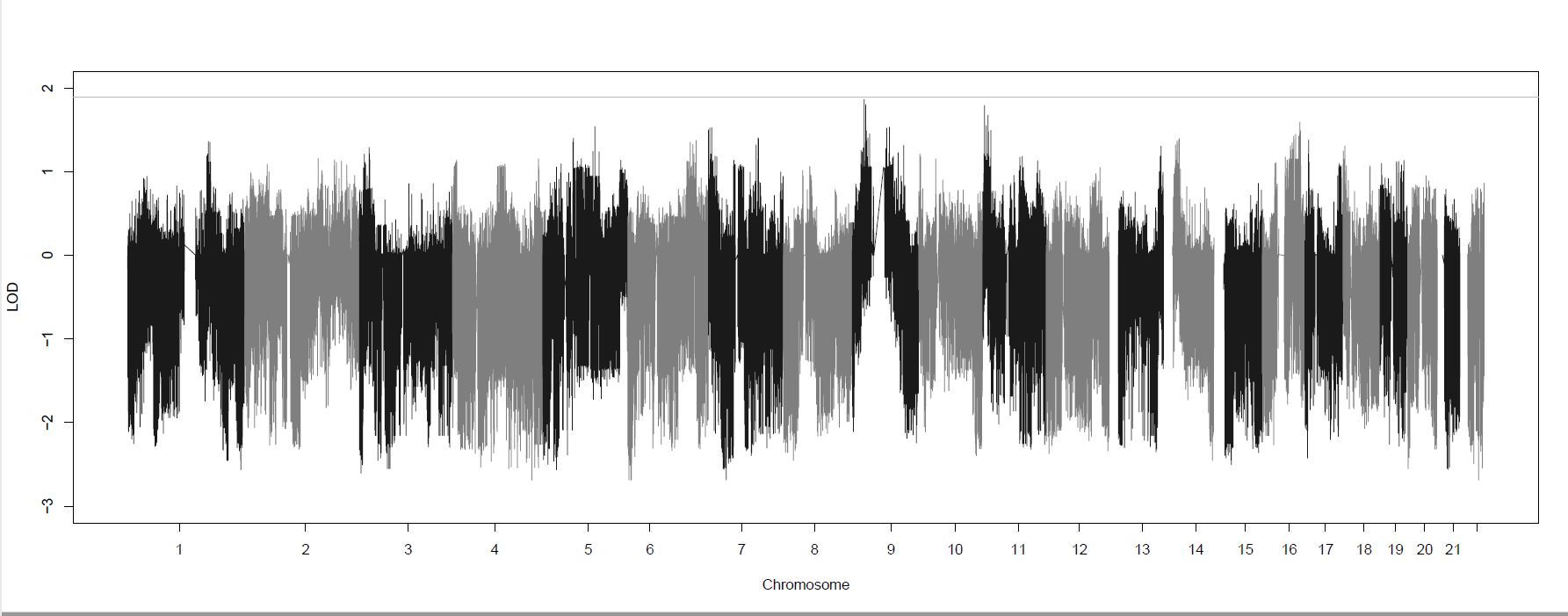


Figure S7:Plot of two-point LOD scores for Family 3

Figure S8: Plot of two-point LOD scores for Family 4
