# Supplementary material for: Genome-wide linkage search for cancer susceptibility loci in a cohort of non BRCA1/2 families in Sri Lanka
Source: BMC Res Notes. 2022 Jun 2;15:190. doi: 10.1186/s13104-022-06081-5 (PMC9164366; doi:10.1186/s13104-022-06081-5)
Supplement: Supplementary file 2 — Additional file 2: Table S1. Details of the cancer in affected relatives of the breast cancer index patient in each family. Table S2. The health status and the number of family members who provided biospecimens for genotyping. Table S3. A description of top LOD scores of family 1. Table S4. A description of top LOD scores of family 2. Table S5. A description of top LOD scores of family 3. Table S6. A description of top LOD scores of family 4. [file 13104_2022_6081_MOESM2_ESM.docx]

| **ID** | **Age of index case at diagnosis** | **1^st^ degree cancer affected cases** | **2^nd^ degree cancer affected cases** | **3^rd^ degree cancer affected cases** | **4^th^ degree cancer affected cases** |
| --- | --- | --- | --- | --- | --- |
| **1** | 43 | Breast CA | Breast CA | - |  |
| **2** | 52 | Breast CA, Melanoma | Thyroid CA | Breast CA |  |
| **3** | 34 | Uterine CA Colorectal CA | Colorectal CA Breast CA Uterine CA | Colorectal CA (2) Throat CA Uterine CA | Breast CA Eye CA |
| **4** | 42 | Breast CA (2) | Breast CA | Breast CA |  |

Supplemental Table S1:Details of the cancer in affected relatives of the breast cancer index patient in each family

Supplemental Table S2: The health status and the number of family members who provided biospecimens for genotyping

| **Family ID** | **Breast CA cases** | **Other CA cases** | **Healthy individuals** | **WES sequenced cases** | **Genotyped affected individuals** | **Genotyped healthy individuals** |
| --- | --- | --- | --- | --- | --- | --- |
| **1** | 03 | 0 | 06 | 1 | 03 | 06 |
| **2** | 03 | 03 | 07 | 1 | 06 | 07 |
| **3** | 02 | 02 | 15 | 0 | 04 | 15 |
| **4** | 02 | 0 | 04 | 1 | 02 | 04 |

Supplemental Table S3: A description of top LOD scores of family1

| Chromosome | Position | ID | LOD.FAM1 | Funtion | Gene |
| --- | --- | --- | --- | --- | --- |
| 1 | 221688.6 | rs114851469 | 0.5422 | exonic | *HSPG2* |
| 1 | 2116546 | GSA-rs34049451 | 0.5422 | exonic | *RD3* |
| 5 | 1492124 | GSA-rs45520937 | 0.5422 | exonic | *PPARGC1B* |
| 5 | 1600975 | rs958912 | 0.5422 | exonic | *ATP10B* |
| 6 | 1169440 | GSA-rs41289942 | 0.5422 | exonic | *RSPH4A* |
| 16 | 536717.5 | rs2111119 | 0.5422 | exonic | *RPGRIP1L* |
| 17 | 16114.33 | rs4790809 | 0.5422 | exonic | *TLCD2* |
| 18 | 441577.6 | GSA-rs34589386 | 0.5422 | exonic | *LOXHD1* |
| 19 | 489544.2 | rs2302951 | 0.5422 | exonic | *GRWD1* |
| 1 | 2227320 | GSA-rs76003336 | 0.5418 | exonic | *TAF1A* |
| 19 | 125412.5 | GSA-rs45531232 | 0.5415 | exonic | *ZNF443* |
| 17 | 671499.7 | rs10491178 | 0.5409 | exonic | *ABCA10* |
| 5 | 790247.8 | rs16877109 | 0.5331 | exonic | *CMYA5* |
| 8 | 198197.2 | rs328 | 0.5331 | exonic | *LPL* |
| 17 | 738264.9 | GSA-rs35037984 | 0.5331 | exonic | *UNC13D* |
| 12 | 1254346 | rs4447263 | 0.5241 | exonic | *DHX37* |
| 17 | 563966.1 | rs2072145 | 0.5241 | exonic | *TSPOAP1* |
| 17 | 564050.2 | rs61743272 | 0.5241 | exonic | *TSPOAP1* |
| 19 | 158530.8 | rs2240229 | 0.5241 | exonic | *OR10H3* |
| 12 | 1230308 | rs7968222 | 0.5241 | exonic | *KNTC1* |
| 16 | 105748 | GSA-rs9932051 | 0.5241 | exonic | *ATF7IP2* |
| 16 | 105748.1 | GSA-rs9931441 | 0.5241 | exonic | *ATF7IP2* |
| 16 | 105758.2 | GSA-rs1970817 | 0.5241 | exonic | *ATF7IP2* |
| 15 | 582561.3 | GSA-rs4646626 | 0.5241 | exonic | *ALDH1A2* |
| 12 | 1222779 | rs36023382 | 0.5173 | exonic | *HPD* |
| 1 | 157021.3 | GSA-rs17368978 | 0.5152 | exonic | *FHAD1* |
| 19 | 454120.8 | rs7412_10AT | 0.5152 | exonic | *APOE* |
| 19 | 454120.8 | rs7412_15AT | 0.5152 | exonic | *APOE* |
| 19 | 454123.4 | rs190853081 | 0.5152 | exonic | *APOE* |
| 9 | 1362876 | GSA-rs34024143 | 0.5152 | exonic | *ADAMTS13* |
| 5 | 1154284 | GSA-rs1129495 | 0.5152 | exonic | *COMMD10* |
| 16 | 159318.2 | rs113363750 | 0.5152 | exonic | *MYH11* |
| 1 | 255729.8 | GSA-rs34484514 | 0.5152 | exonic | *RSRP1* |
| 1 | 180233.7 | GSA-rs2270978 | 0.515 | exonic | *ARHGEF10L* |
| 1 | 180236.9 | GSA-rs2270976 | 0.515 | exonic | *ARHGEF10L* |
| 1 | 2304151 | rs2273970 | 0.5139 | exonic | *GALNT2* |
| 9 | 789102.4 | GSA-rs2842480 | 0.5139 | exonic | *PCSK5* |
| 12 | 1222479 | rs61736010 | 0.5112 | exonic | *SETD1B* |
| 17 | 594897.1 | GSA-rs77617620 | 0.5111 | exonic | *C17orf82* |

Supplemental Table S4: A description of top LOD scores of family2

| Chromosome | Position | ID | LOD.FAM2 | Function | GENE |
| --- | --- | --- | --- | --- | --- |
| 2 | 1556290 | GSA-rs12616962 | 1.3947 | intronic | *KCNJ3* |
| 2 | 1532999 | rs10179418 | 1.3941 | intronic | *FMNL2* |
| 2 | 1512865 | GSA-rs12996561 | 1.3936 | intergenic | *LINC01817;RND3* |
| 2 | 1540325 | rs892747 | 1.3935 | intergenic | *ARL6IP6;RPRM* |
| 2 | 1533034 | GSA-rs115714536 | 1.3935 | intronic | *FMNL2* |
| 2 | 1569358 | GSA-rs10932860 | 1.3935 | ncRNA_intronic | *LINC01876* |
| 2 | 1532817 | rs4664583 | 1.3934 | intronic | *FMNL2* |
| 2 | 1570308 | rs2049582 | 1.3934 | ncRNA_intronic | *LINC01876* |
| 22 | 486981.2 | rs133620 | 1.3933 | intergenic | *MIR3201;FAM19A5* |
| 2 | 1531698 | GSA-rs114519242 | 1.3917 | intergenic | *STAM2;FMNL2* |
| 13 | 1136758 | GSA-rs9577426 | 1.3878 | intronic | *MCF2L* |
| 20 | 100921.6 | GSA-rs6133801 | 1.3858 | ncRNA_intronic | *SNAP25-AS1* |
| 6 | 1135412 | rs9400601 | 1.3852 | intergenic | *RFPL4B;LOC101927686* |
| 6 | 1135450 | rs796495 | 1.3852 | intergenic | *RFPL4B;LOC101927686* |
| 13 | 367148.1 | rs1410641 | 1.3851 | intergenic | *DCLK1;CCDC169-SOHLH2* |
| 20 | 66536.23 | rs11698104 | 1.3847 | intergenic | *CASC20;LINC01713* |
| 2 | 1423924 | rs10496890 | 1.3841 | intronic | *LRP1B* |
| 2 | 1337880 | rs17733147 | 1.384 | intronic | *NCKAP5* |
| 2 | 1591309 | rs1521873 | 1.384 | intronic | *CCDC148* |
| 20 | 85430 | GSA-rs117704944 | 1.3833 | intronic | *PLCB1* |
| 20 | 111749 | rs2207418 | 1.3831 | intergenic | *C20orf187;LOC339593* |
| 2 | 1350258 | rs16830300 | 1.3827 | intronic | *MGAT5* |
| 13 | 437009.2 | rs2325023 | 1.3827 | ncRNA_intronic | *LINC00400* |
| 13 | 1137172 | rs9577454 | 1.3812 | intronic | *MCF2L* |
| 20 | 68589.14 | rs6038631 | 1.3805 | intergenic | *BMP2;LINC01428* |
| 20 | 110708.9 | rs62191644 | 1.3803 | intergenic | *C20orf187;LOC339593* |
| 22 | 487265.2 | GSA-rs909497 | 1.3793 | intergenic | *MIR3201;FAM19A5* |
| 20 | 105748.6 | rs78118899 | 1.3792 | intronic | *SLX4IP* |
| 22 | 487471.2 | rs13054685 | 1.3788 | intergenic | *MIR3201;FAM19A5* |
| 13 | 1125052 | rs4293272 | 1.3785 | intergenic | *LINC02337;LINC00354* |
| 20 | 68390.06 | rs16992673 | 1.3782 | intergenic | *BMP2;LINC01428* |
| 2 | 1343495 | rs17817136 | 1.378 | intergenic | *NCKAP5;MIR3679* |
| 13 | 1123530 | rs9522363 | 1.3776 | intergenic | *LINC02337;LINC00354* |
| 13 | 1116620 | rs8001852 | 1.3765 | intergenic | *LINC00431;LINC00368* |
| 2 | 1458053 | GSA-rs78809153 | 1.3765 | ncRNA_intronic | *TEX41* |
| 13 | 1125255 | GSA-rs79752671 | 1.3763 | intergenic | *LINC02337;LINC00354* |
| 20 | 83940.6 | GSA-rs74183555 | 1.3754 | intronic | *PLCB1* |
| 2 | 1424598 | GSA-rs74598322 | 1.375 | intronic | *LRP1B* |
| 20 | 108235.1 | rs6134049 | 1.3742 | intergenic | *LINC01752;LOC101929413* |
| 13 | 384406.5 | rs17273171 | 1.3738 | intronic | *TRPC4* |
| 22 | 489307.8 | GSA-rs73888446 | 1.3725 | intronic | *FAM19A5* |
| 20 | 108977.9 | rs6108744 | 1.3724 | intergenic | *LOC101929413;C20orf187* |
| 13 | 1131102 | rs75722751 | 1.3717 | intergenic | *SPACA7;TUBGCP3* |
| 2 | 1575394 | GSA-rs16840676 | 1.3711 | intergenic | *GPD2;GALNT5* |
| 13 | 405017.7 | rs1555627 | 1.3701 | intergenic | *COG6;LINC00332* |
| 2 | 1332193 | GSA-rs72845872 | 1.369 | intronic | *GPR39* |
| 2 | 1415234 | GSA-rs113443790 | 1.3685 | intronic | *LRP1B* |
| 2 | 1623618 | GSA-rs145260432 | 1.3457 | ncRNA_exonic | *AHCTF1P1* |
| 13 | 1111603 | rs391859 | 1.3181 | exonic | *COL4A2* |

Supplemental Table S5: A description of top LOD scores of family3

| Chromosome | Position | ID | LOD.FAM3 | Function | Gene |
| --- | --- | --- | --- | --- | --- |
| 9 | 251777.2 | GSA-rs1925508 | 1.867 | intergenic | *IZUMO3;TUSC1* |
| 9 | 282281.7 | GSA-rs10812758 | 1.8037 | intronic | *LINGO2* |
| 11 | 38913.8 | rs11825543 | 1.7952 | intronic | *STIM1* |
| 11 | 113739.6 | rs2071461 | 1.6787 | exonic | *CSNK2A3* |
| 16 | 796870 | GSA-rs889791 | 1.5952 | intergenic | *MAF;MAFTRR* |
| 16 | 796936.7 | rs4888039 | 1.5952 | intergenic | *MAF;MAFTRR* |
| 16 | 796889.3 | GSA-rs8048408 | 1.5879 | intergenic | *MAF;MAFTRR* |
| 11 | 113755.7 | rs7937567 | 1.5648 | intronic | *GALNT18* |
| 11 | 76161.91 | rs9787734 | 1.554 | intronic | *PPFIBP2* |
| 5 | 1108252 | rs42670 | 1.5447 | UTR3 | *CAMK4* |
| 9 | 787862.2 | GSA-rs17062158 | 1.5365 | intronic | *PCSK5* |
| 11 | 76020.72 | rs11827218 | 1.5347 | intronic | *PPFIBP2* |
| 7 | 73449.65 | GSA-rs112069447 | 1.53 | intergenic | *LOC101927354;COL28A1* |
| 7 | 32261.29 | rs12700671 | 1.5258 | intergenic | *LOC100129603;SDK1* |
| 9 | 731832.8 | GSA-rs10746850 | 1.5234 | intronic | *TRPM3* |
| 7 | 3852.06 | rs139835390 | 1.5008 | intergenic | *FAM20C;LOC442497* |

Supplemental Table S6: A description of top LOD scores of family4

| Chromosome | Position | ID | LOD.FAM4 | Function | Gene |
| --- | --- | --- | --- | --- | --- |
| 9 | 53354.7 | GSA-rs2273782 | 0.7625 | exonic | *RLN1* |
| 12 | 1333784 | rs2291258 | 0.7625 | exonic | *GOLGA3* |
| 2 | 1410815 | rs139908062 | 0.7624 | exonic | *LRP1B* |
| 16 | 558669.3 | 16:55866934 | 0.7623 | exonic | *CES1* |
| 10 | 981556.8 | rs41291628 | 0.7623 | exonic | *TLL2* |
| 16 | 772433.3 | GSA-rs61740088 | 0.7623 | exonic | *SYCE1L* |
| 4 | 795074.2 | GSA-rs41278059 | 0.7623 | exonic | *ANXA3* |
| 2 | 711631.7 | rs17720303 | 0.7623 | exonic | *ATP6V1B1* |
| 3 | 449744.3 | GSA-rs3087823 | 0.7623 | exonic | *ZDHHC3* |
| 2 | 540815.5 | GSA-rs34000641 | 0.7623 | exonic | *GPR75* |
| 3 | 578826 | rs17058639 | 0.7623 | exonic | *SLMAP* |
| 2 | 1219929 | GSA-rs201046687 | 0.7623 | exonic | *TFCP2L1* |
| 1 | 1566402 | rs3748570 | 0.7302 | exonic | *NES* |
| 3 | 369407.4 | GSA-rs9864910 | 0.7302 | exonic | *TRANK1* |
| 3 | 449433.9 | rs1995641 | 0.7302 | exonic | *TGM4* |
| 3 | 499490.7 | GSA-rs868891 | 0.7302 | exonic | *MON1A* |
| 3 | 524296.7 | rs419752 | 0.7302 | exonic | *DNAH1* |
| 6 | 1467204 | GSA-rs41305288 | 0.723 | exonic | *GRM1* |
